# *In Vivo* Toxicity Evaluation of PEGylated CuInS_2_/ZnS Quantum Dots in BALB/c Mice

**DOI:** 10.3389/fphar.2019.00437

**Published:** 2019-04-25

**Authors:** Wenyi Zou, Li Li, Yajing Chen, Tingting Chen, Zhiwen Yang, Jie Wang, Dongmeng Liu, Guimiao Lin, Xiaomei Wang

**Affiliations:** ^1^College of Life Sciences and Oceanography, Shenzhen University, Shenzhen, China; ^2^Department of Physiology, School of Basic Medical Sciences, Shenzhen University Health Science Center, Shenzhen University, Shenzhen, China

**Keywords:** CuInS_2_/ZnS quantum dots, nanotoxicology, non-cadmium, toxicity, *in vivo*

## Abstract

In recent years, quantum dots (QDs) have emerged as a potential contrast agent for bioimaging due to their bright luminescence and excellent photostability. However, the wide use of QDs *in vivo* has been limited due to underlying toxicity caused by leakage of heavy metals. Although non-cadmium QDs have been developed to resolve this issue, a comprehensive understanding of the toxicity of these newly developed QDs remains elusive. In this study, we administered PEGylated copper indium sulfide/zinc sulfide (CuInS_2_/ZnS), which are typical non-cadmium QDs, and analyzed the long-term effects of these nanoparticles in BALB/c mice. Body weight, hematology, blood biochemistry, organ histology, and biodistribution were examined at different time points. We found no significant difference in body weight after injection of CuInS_2_/ZnS QDs. These CuInS_2_/ZnS QDs entered and were accumulated in major organs for 90 days post-injection. The majority of biochemical indicators were not significantly different between the QDs-treated group and the control group. In addition, no significant histopathological abnormalities were observed in the treated mice compared with the control mice. CuInS_2_/ZnS QDs did not lead to observable toxicity *in vivo* following either the administration of a high or low dose. Our research not only provides direct evidence of the bio-safety of CuInS_2_/ZnS QDs, but also a feasible method for evaluating nanoparticle toxicity.

## Introduction

Quantum dots (QDs) are semiconductor nanomaterials composed of a finite number of atoms, are three-dimensional in the order of nanometers, and include CdSe, CdTe, and PbS (Veeranarayanan et al., 2012). QDs have many attractive optical properties, such as strong fluorescent intensity, wide excitation spectra, narrow emission spectra, resistance to photobleaching, and strong stability. QDs are widely used in bioimaging, drug delivery, optical sensing, and biological labeling (Lin et al., 2015b; Peng et al., 2018; Zhou et al., 2018). However, with the increasing use of QDs, there is concern regarding the potential impact of QDs on the environment and humans. A number of studies have shown that QDs are distributed in major organs and can accumulate in some tissues for a long period (Li et al., 2016). Many cell lines can take up QDs, which leads to damage such as reduced cell viability, induction of apoptosis, oxidative stress, and cell morphology changes (Chen et al., 2018).

Although the toxicity of QDs has been demonstrated, to some extent, most previous QDs cytotoxicity studies focused on cadmium-based nanocrystals (Ho et al., 2013; Liu et al., 2013). It is known that cadmium can cause adverse health effects when cadmium-based QDs enter the body. Cho et al. (2007) found that cadmium ions are released from CdSe QDs, and induce cytotoxicity by mechanisms such as intracellular reactive oxygen species production. To reduce the health risks caused by cadmium release from cadmium-containing QDs, non-cadmium QDs such as InP/ZnS, PbSe, and CuInS_2_ have been developed. CuInS_2_ QDs have proved promising as fluorescent probes in *in vivo* imaging due to their excellent fluorescence. To increase their biocompatibility, a “cap” or “shell” of CuInS_2_ is normally designed. ZnS is usually used as a “cap” or “shell” to protect the core element in case of leakage of the toxic core element, making the CuInS_2_/ZnS QDs brighter and more stable than the core-only CuInS_2_ QDs (Pons et al., 2010). Although CuInS_2_/ZnS is a promising nanomaterial, little data regarding the toxicity of CuInS_2_/ZnS are available. For example, Liu et al. (2017) found that exposure to CuInS_2_/ZnS QDs in rare minnow embryos led to developmental toxicity via the induction of oxidative stress and DNA damage, which indicated that CuInS_2_/ZnS QDs were toxic to *Gobiocypris rarus* embryos. A study showed that CuInS_2_ and CuInS_2_/ZnS QDs were non-toxic to HeLa and OECM-1 cells even when the concentration of QDs reached 250 μg/mL (Chen et al., 2015). Another study showed that CuInS_2_/ZnS QDs caused less inflammation than CdTeSe/CdZnS QDs (Thomas et al., 2010). Although the ZnS coating can reduce the toxicity of CuInS_2_ QDs, it cannot completely prevent the adverse effects caused by the nanoparticles themselves (Song et al., 2013; Tang S. et al., 2013). To date, little is known about the toxicity of CuInS_2_/ZnS QDs, especially their long-term toxicity, which in turn hinders the development of their future bioapplications. Therefore, it is necessary to further evaluate the long-term *in vivo* toxicity of CuInS_2_/ZnS QDs.

In this study, we systematically investigated the *in vivo* long-term toxicity of CuInS_2_/ZnS QDs terminated with polyethylene glycol (PEG) functional groups, which are normally used to modify the surface of nanoparticles in order to increase their *in vivo* biocompatibility. In brief, BALB/c mice were administered PEGylated CuInS_2_/ZnS QDs at 2.5 mg/kg or 25 mg/kg intravenously, and then body weight, hematology, blood biochemistry, organ histology, and biodistribution were monitored over a 90-day period. We found that the fluorescent signals from CuInS_2_/ZnS QDs were detectable in the spleen and liver over the 90-day period. There was no obvious change in body weight in the experimental group compared to the control group. The majority of biochemical indicators showed no significant differences between the QDs-treated group and the control group. Moreover, hematological analysis and hematoxylin and eosin (H&E) staining showed no change in the QDs-treated group compared to the control group. In summary, we found that PEGylated CuInS_2_/ZnS QDs did not lead to observable toxicity *in vivo* although they accumulated in the body for a long period. Our research suggested that CuInS_2_/ZnS QDs were highly biocompatible *in vivo* if an appropriate surface modification and dosage were used.

## Materials and Methods

### Characterization of CuInS_2_/ZnS QDs

PEGylated CuInS2/ZnS QDs were purchased from Najingtech Company, Hangzhou city, China. The ratio of Cu: In: Zn is 1:1.8: 25.2 which was determined by ICP-MS. The molecular weight of PEG chain is 2000 Da, and the density of the PEG chains is 0.8–0.9. The optical density of the 10 mg/mL QD solution used was 0.803 at the wavelength of 550 nm. The hydrodynamic size distribution, particle size distribution and zeta potential of the PEGylated CuInS_2_/ZnS QDs were measured using a particle size analyzer (Brookhaven Instruments 90 Plus, United States). The morphological images of CuInS_2_/ZnS QDs were obtained in Wuhan University using a JEOL model Jem-2100 transmission electron microscope (TEM) with an acceleration voltage of 200 kV. Absorption spectra were measured by UV-visible-near-infrared spectrophotometer (TP-720,Tianjin city, China). The photoluminescence emission spectra were measured using a fluorescence spectrophotometer (F-4600, Hitachi, Japan) with an excitation wavelength of 380 nm.

### Animal Injections and Weight Measurements

Male BALB/c mice of 7 weeks old were purchased from the Medical Laboratory Animal Center of Guangdong Province and were housed six per cage at a 12 h/12 h light/dark cycle. The animal experiments were conducted in accordance with the Guide for the Care and Use of Laboratory Animals Center of Shenzhen University and approved by the Experimental Animal Ethics Committee of Shenzhen University (Permit No. 201512022). Each mouse was injected with 100 μL of buffered saline or buffered QD dispersion via the tail vein. Following these injections, the mice were weighed at various time points from 0 to 90 days.

### Frozen Sections and Histology

At 1, 3, 7, 14, 30, 60, and 90 days post-injection, the mice in each group were sacrificed. The organs (heart, liver, spleen, lung, kidney, brain, and testis) were harvested. Frozen sections were obtained using a freezing microtome (CM3050S, Leica, Germany). Fluorescent images of these organs were obtained using a fluorescence microscope (Axio Observer, Zeiss, Germany).

Following organ harvesting, the organs were fixed in 10% formalin for 24 h, and then dehydrated with different gradient concentrations of alcohol. Before staining with H&E, the tissues were embedded in paraffin and sectioned. After staining, the stained slices were observed under a fluorescence microscope (Axio Observer, Zeiss, Germany).

### Inductively Coupled Plasma Mass Spectrometry (ICP-MS) Analysis

Before ICP-MS analysis, the major organs (heart, liver, spleen, lung, kidney, brain, and testis) were digested with 4 mL 65% HNO_3_ and 1 mL 30% H_2_O_2_ for 20 min at 180°C using microwave acid digestion apparatus (Milestone ETHOS ONE). In order to reduce the concentration of HNO_3_, the solution was diluted 10 times with deionized water after digestion. The concentration of In element in the sample was tested by ICP-MS (Nexlon 300X, PerkinElmer, United States).

### Routine Blood Examination and Serum Biochemistry

After injection, blood samples from each group were obtained on day 1, 3, 7, 14, 30, 60, and 90. Routine blood examination was conducted using a blood analyzer (Dymind, D5-CRP, China). To determine serum biochemistry, indicators of kidney, liver, and heart function were measured by a blood biochemistry analyzer (Mindray, BS-220, China).

### Statistical Analysis

All data are presented as mean ± standard deviation (SD). Statistical analysis was performed using the SPSS 11.0 package. Analyses of differences between the experimental groups were performed by one-way ANOVA. Differences were considered significant at *p* < 0.05 and extremely significant at *p* < 0.01.

## Results

### Characterization of QDs

**FIGURE 1:**
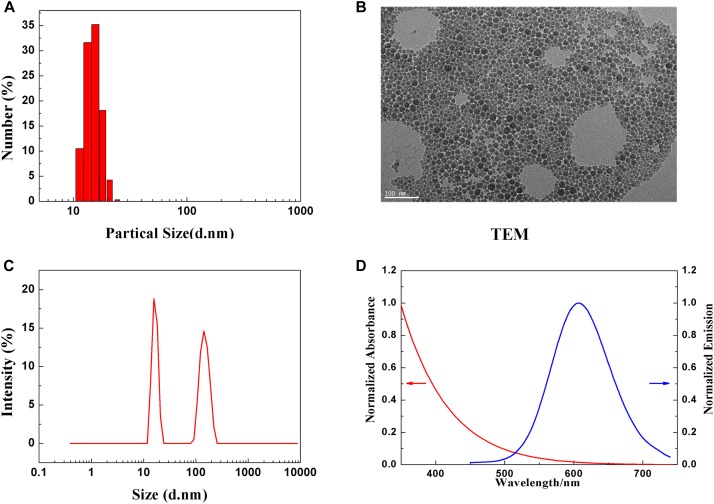
Characterization of PEGylated CuInS_2_/ZnS QDs. **(A)** Hydrodynamic size distribution. **(B)** Representative TEM image of CuInS_2_/ZnS QDs. **(C)** Particle size distribution. **(D)** Normalized absorption (red line) and PL emission spectra (blue line).

Dynamic light scattering was used to measure the hydrodynamic diameter and zeta potential of CuInS_2/_ZnS QDs. Figure 1A shows that the average hydrodynamic diameter of CuInS_2_/ZnS QDs was approximately 13–15 nm. Figure 1B shows a representative transmission electron microscopy (TEM) image of CuInS_2_/ZnS QDs. The TEM size distribution was approximately 10 nm. The particle size distribution weighted by intensity indicated slight agglomeration of QD in solution (Figure 1C). The zeta potential of the CuInS_2_/ZnS QDs was approximately −13.52 mV. Figure 1D shows the extinction and photoluminescence spectra of the CuInS_2_/ZnS QDs. The emission peak of QDs was around 610 nm.

### Body Weight

**FIGURE 2:**
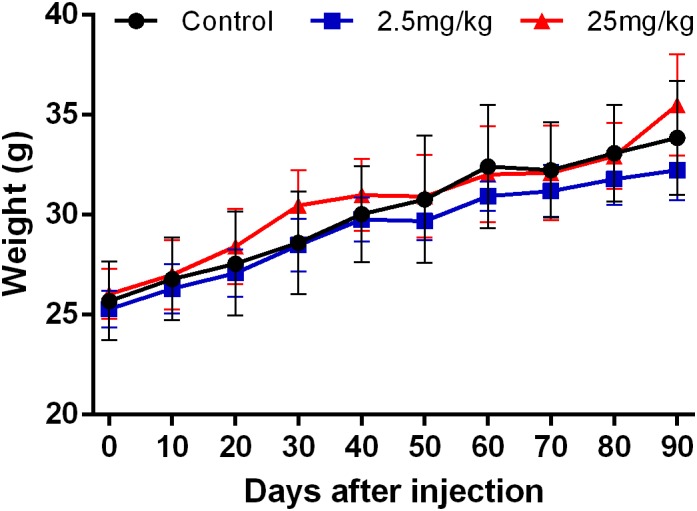
The body weight of mice was monitored after injection of the QD/buffer solution.

To evaluate the effect of CuInS_2_/ZnS QDs on the development and growth of mice, body weight was continuously measured. As shown in Figure 2, there were no significant differences in the average body weight after injection of CuInS_2_/ZnS QDs over the 90-day period, in either the high dose QDs-treated group or the low dose-treated group. In addition, the behavior and appearance of mice after QDs injection were continuously monitored. No significant differences in eating, drinking, hair color, and glossiness were found between the QDs-treated mice and the control mice. No aggression or unusual responses were observed in treated mice compared to control mice. These results showed that the CuInS_2_/ZnS QDs were biocompatible in mice.

### Fluorescent Images of Major Organs

**FIGURE 3:**
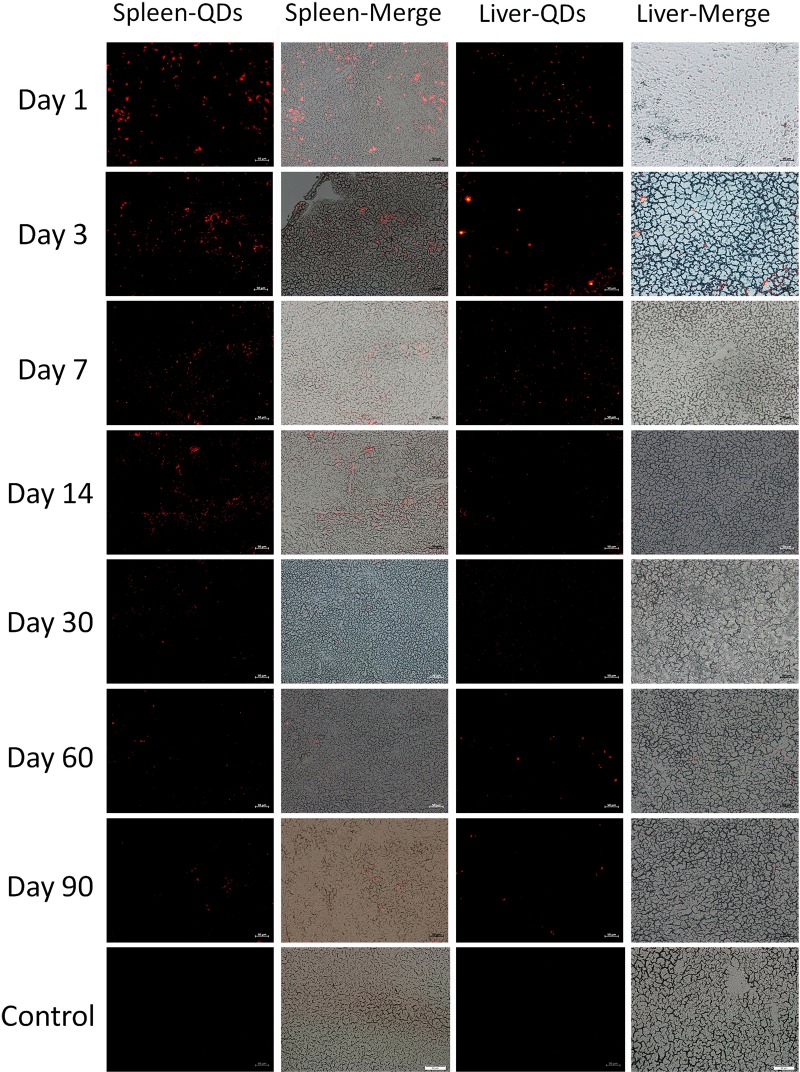
Fluorescence images of the spleen and liver of treated mice at different time points. Scale bar represents 50 μm.

In order to study the distribution of CuInS_2_/ZnS QDs in mice, the major organs including heart, liver, lung, spleen, kidney, and brain were sliced into 5 μm sections for fluorescence imaging at different time points. As shown in Figure 3, the results showed that the QDs emitted strong fluorescence in the liver and spleen up to 90 days after injection. In addition, a few fluorescent signals were observed in the lung and kidney up to 30 days after injection (Supplementary Figures S1, S2). These results suggested that the QDs with fluorescence emitting ability are taken up by the spleen and liver and accumulated over 90 days.

### Inductively Coupled Plasma Mass Spectrometry (ICP-MS) Analysis

**FIGURE 4:**
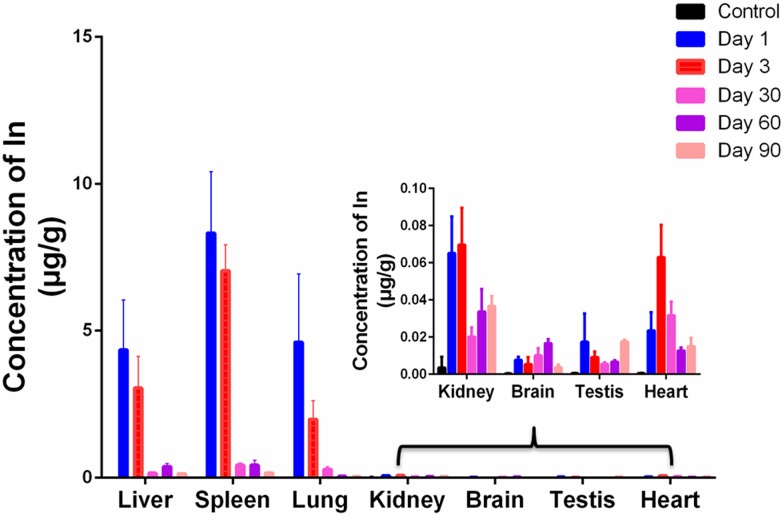
ICP-MS analysis of the major organs at various time points. Values are the means ± SD, *n* = 3.

To further determine the accumulation and biodistribution of CuInS_2_ in mice, the concentrations of indium in the major organs were measured by ICP-MS. We first performed ICP-MS analysis to calculate concentrations of Cu, In and Zn. Our results showed that the CuInS_2_/ZnS QDs solution we injected corresponded to 0.21 mg/kg of Cu, 0.68 mg/kg of In, and 5.37 mg/kg of Zn. Figure 4 shows that indium from QDs was mainly accumulated in the liver, spleen, and lung after injection. On day 1, the concentrations of indium in the liver, spleen, and lung were 4.350 ± 1.699 μg/g, 8.322 ± 2.094 μg/g, and 4.607 ± 2.329 μg/g, respectively. The indium concentration *in vivo* gradually decreased with time. On day 90, the concentrations of indium in the liver, spleen, and lung were 0.130 ± 0.022 μg/g, 0.158 ± 0.038 μg/g, and 0.029 ± 0.004 μg/g, respectively. Indium was still detected in the kidney, heart, brain, and testis 90 days after injection. This suggested that indium from QDs can remain in the major organs for up to 90 days.

### Routine Blood Examination

**FIGURE 5:**
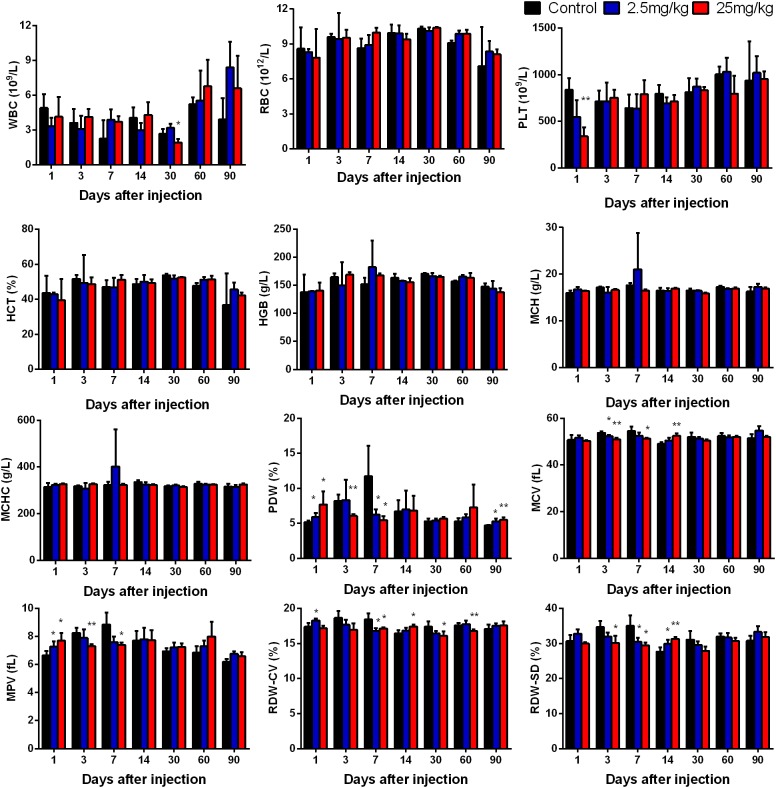
Hematology markers were monitored over the 90-day treatment period. Abbreviations: white blood cell count, WBC; red blood cell count, RBC; platelet, PLT; hematocrit, HCT; hemoglobin, HGB; mean corpuscular hemoglobin, MCH; mean corpuscular hemoglobin concentration, MCHC; platelet distribution width, PDW; mean corpuscular volume, MCV; mean platelet volume, MPV; coefficient of variation of RBC volume distribution width, RDW-CV; standard deviation of RBC volume distribution width, RDW-SD. Values are the means ± SD, *n* = 5. Values significantly different from the control are indicated by asterisks (^∗^*p* < 0.05; ^∗∗^*p* < 0.01).

When QDs enter the body through a vein, they first react with the cells and components in the blood. To evaluate the effect of CuInS_2_/ZnS QDs on blood cells, a routine blood examination was carried out. White blood cell count (WBC), red blood cell count (RBC), Platelet (PLT) count, hematocrit (HCT), hemoglobin (HGB) level, mean corpuscular hemoglobin (MCH), mean corpuscular hemoglobin concentration (MCHC), platelet distribution width (PDW), mean corpuscular volume (MCV), mean platelet volume (MPV), coefficient of variation of RBC volume distribution width (RDW-CV), and standard deviation of RBC volume distribution width (RDW-SD) were determined. Figure 5 demonstrates that the experimental group showed significant differences in some biochemical indicators at some time points compared with the control group; however, most of the biochemical indicators did not differ significantly at final time point (day 90), with the exception of PDW. This suggested that CuInS_2_/ZnS QDs did not cause obvious adverse effects in blood cells.

### Clinical Biochemistry

**FIGURE 6:**
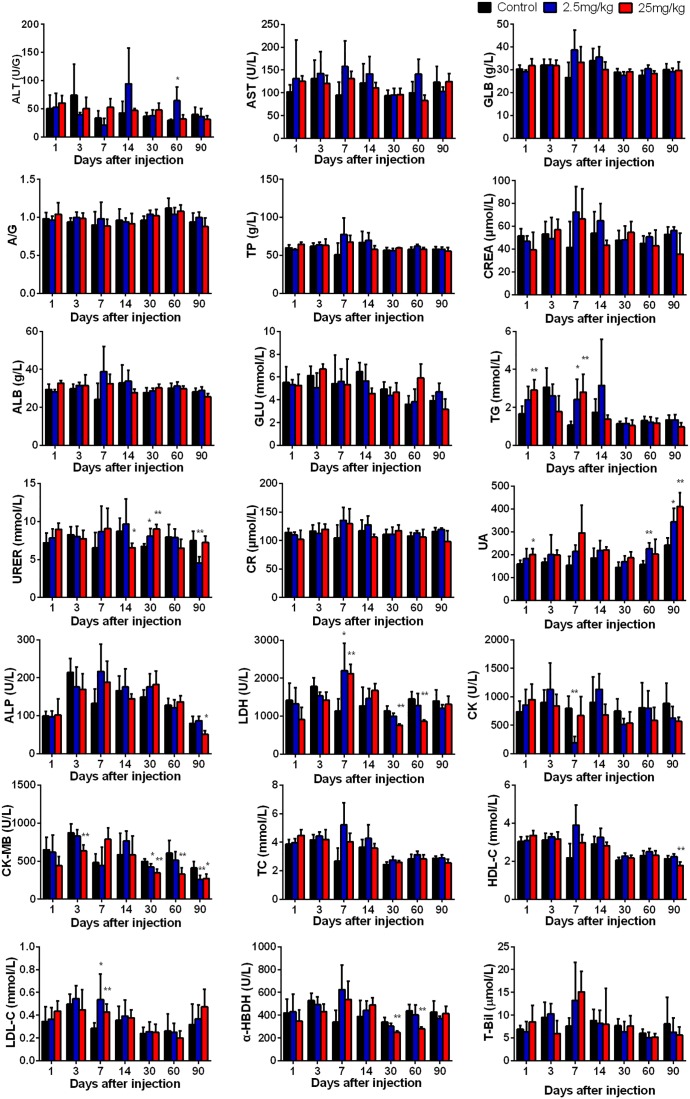
The serum biochemical biomarkers were measured at the indicated time points after treatment. Abbreviations: alanine aminotransferase, ALT; aspartate transaminase, AST; the ratio of albumin and globulin, A/G; globulin, GLB; total protein, TP; albumin, ALB; glucose, GLU; triglyceride, TG; urea, UREA; creatinine CR; creatinine minus sixty-three, CREA; uric acid, UA; lactate dehydrogenase, LDH; alkaline phosphatase, ALP; creatine kinase, CK; creatine kinase-MB, CK-MB; total cholesterol, TC; high-density lipoprotein cholesterol, HDL-C; low-density lipoprotein cholesterol, LDL-C; α-hydroxybutyrate dehydrogenase, α-HBDH. Values are the means ± SD, *n* = 5. Values significantly different from the control are indicated by asterisks (^∗^*p* < 0.05; ^∗∗^*p* < 0.01).

In order to investigate the effect of CuInS_2_/ZnS QDs accumulation on the functions of major organs, we determined the physiological function of these organs by measuring a series of serum biomarkers. Indicators of liver function included glucose (GLU), total cholesterol (TC), alanine aminotransferase (ALT), aspartate transaminase (AST), triglyceride (TG), low-density lipoprotein cholesterol (LDL-C), albumin (ALB), alkaline phosphatase (ALP) and high-density lipoprotein cholesterol (HDL-C). Indicators of kidney function included uric acid (UA), total protein (TP), and urea (UREA). Indicators of heart function included creatine kinase (CK), creatinine (CREA), creatine kinase-MB (CK-MB), lactate dehydrogenase (LDH), and α-hydroxybutyrate dehydrogenase (α-HBDH). In addition, we determined the content of total bilirubin (T-Bil) in the serum. As we know, T-Bil not only can reflect the function of liver, but also reflect whether hemolysis occurs in the body. From Figure 6, it can be seen that no obvious differences were found between the QDs-treated group and the control group in the majority of serum biomarkers at final time point (day 90), except for UREA, UA, CK-MB, ALP, and HDL-C. These results indicated that QDs affected the content of some serum components, but did not lead to functional damage due to the body’s complexity and resistance.

### Histological Changes

**FIGURE 7:**
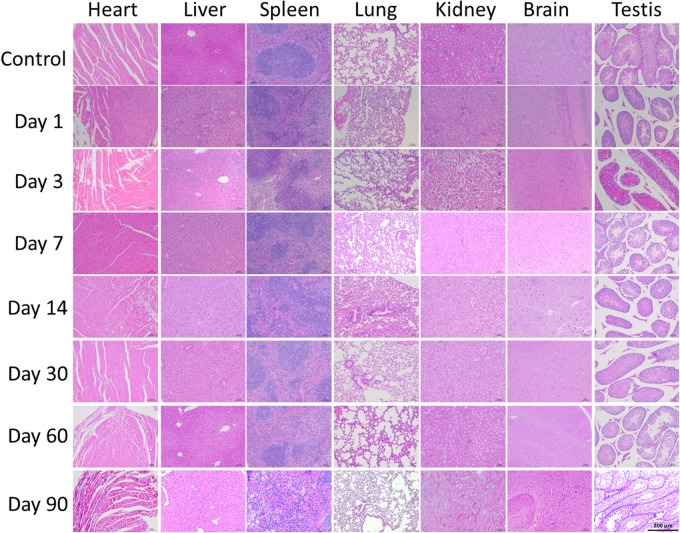
Representative histological images of murine major organs at different time points after intravenous injection of CuInS_2_/ZnS QDs. Scale bar represents 50 μm.

As the QDs accumulated in major organs, we wondered if the QDs would cause histomorphic adverse effects in these organs. The heart, liver, spleen, lung, kidney, brain, and testis were sliced into 5 μm sections and stained with H&E. The results are shown in Figure 7 and demonstrate that no changes were observed in treated mice compared with control mice. Therefore, although the QDs remained in these organs, especially in spleen and liver for 90 days after injection, no significant histopathological abnormalities were observed. These results suggested that PEGylated CuInS_2_/ZnS QDs showed little toxicity in these organs.

## Discussion

To date, QDs have been produced and widely used in many fields such as biomedicine, optics, and physics. Due to the wide application of QDs, there are many ways in which humans are exposed to QDs. Hence, there is growing concern regarding the toxicity of QDs. The majority of toxicity studies on QDs have been based on the *in vitro* cell model (Zhang et al., 2011). Compared with the data obtained *in vitro*, there is insufficient data on *in vivo* toxicity. The potential toxicity of each QD should be evaluated both *in vitro* and *in vivo* as data acquired in and out of the body are often not completely consistent. To our knowledge, the *in vitro* model is simpler than the *in vivo* model, and can eliminate the interference of many complicated factors, which is advantageous in the research of detailed molecular mechanisms accounting for toxicological behavior. However, the *in vitro* model cannot simulate the complexity of the biological process of QDs *in vivo*. Toxicity *in vivo* is influenced by many factors such as dosage, administration route, biodistribution, excretion, elimination by the immune system, and the animal model used. In addition, the physical and chemical properties of QDs such as shape, size, composition, and surface coating can also affect their potential toxicity *in vivo*. For example, Byron et al. found that different surface coatings on QDs determined localization of the QDs *in vivo* (Ballou et al., 2004). Xiao et al. demonstrated that adenosine 5′-monophosphate modified CdSe/ZnS QDs could stimulate the growth of *Tetrahymena thermophila*, while mercaptoacetic acid-capped CdSe/ZnS QDs inhibited growth (Xiao et al., 2012).

In this study, we investigated the long-term toxicity of PEGylated CuInS_2_/ZnS QDs in BALB/c mice at concentrations up to 25 mg/kg. This study provided information for clinical applications and the foundation for the design and development of biologically targeted nanoparticles for biomedical applications.

One of the key indices in evaluating toxicity *in vivo* is the biodistribution of QDs. Our study demonstrated that CuInS_2_/ZnS QDs can accumulate in the spleen and liver for more than 3 months after injection. These results are consistent with a previous report (Ye et al., 2012). In addition, Su et al. (2011) previously reported that aqueous synthesized QDs initially accumulated in the liver a short time after injection. These results also revealed that the breakdown and clearance of QDs are quite slow, suggesting that longer-term studies will be required to examine the ultimate fate of these heavy metals and the influence of their accumulation in mice. Contrary to our results, Choi et al. (2007) found that no accumulation of QDs was observed in the liver of male CD-1 mice after injection. The authors explained that as the size of the QDs was within the kidney filtration size limit, a number of QDs were removed by renal excretion. Although we did not observe QD fluorescence in the brain, kidney, and lung, etc, this does not mean that the QDs had been removed from the organism (Lin et al., 2015a). It is possible that the QDs had broken up and fluorescence of the QDs was unable to be detected.

As the QDs were injected into the tail vein, they were in direct contact with blood cells. The QDs are similar in size to viruses and large proteins, and may influence the immune system or induce an inflammatory response, which would be indicated by changes in hematological factors, such as the WBC (Xiao et al., 2012). Thus, it was essential to investigate the quantity change or shape distribution of blood cells in mice, such as red blood cells and white blood cells. Our findings showed that the blood cells examined were not significantly different between the control group and the experimental group at day 90, with the exception of PLTs, suggesting little toxicity in BALB/c mice.

The liver plays an important role in biotransformation and the major function of the kidney is excretion of foreign substances. When QDs reach the liver and kidney via the blood circulation, this may result in adverse effects on liver and kidney function. Previous studies have proved that it is useful to measure liver function, kidney function, and hepatocellular injury by determining a series of parameters in serum (Dufour et al., 2000). Our results showed that PEGylated CuInS_2_/ZnS QDs did not cause any obvious abnormalities in the serum indicators tested, suggesting that CuInS_2_/ZnS QDs have low toxicity in the body. In addition, although CuInS_2_/ZnS QDs can remain in the spleen and liver for more than 90 days post-injection, they did not induce any pathological injuries to the structure of the major organs as shown by H&E staining. These results indicated that CuInS_2_/ZnS QDs exhibited optimal biocompatibility and had low toxicity in BALB/c mice.

It should be emphasized that the CuInS_2_/ZnS QDs used were PEGylated. PEGylation refers to the modification of a protein, peptide or non-peptide molecule with a PEG chain. These PEG conjugates have the following advantages: increased solubility, improved biocompatibility and biological stability, prolonged residence in the body, decreased degradation by enzymes, and reduced protein immunogenicity (Ikeda and Nagasaki, 2011). Therefore, PEGylation now plays an important role in drug delivery, imaging, and other nanoparticle bioapplications. Nanoparticles are generally unstable and may cause adverse effects in biological organisms; however, coating these nanoparticles with PEG can overcome this problem.

It has been reported that PEG coating not only greatly reduces toxicity, but also shows little interaction with tissue components (Nekolla et al., 2016). Tang et al. utilized QDs with a positive, negative, or a PEG coating to study toxicity in a mouse model, and found that QDs coated with PEG resulted in fewer chronic injuries *in vivo* compared to the positive or negative QDs (Tang Y. et al., 2013). In our study, the QDs used consisted of a CuInS_2_ core and a ZnS shell, coated with PEG. Our results showed that PEGylated CuInS_2_/ZnS QDs did not cause any adverse effects in BALB/c mice for 90 days after injection. In summary, these results showed that the toxicity of PEGylated CuInS_2_/ZnS QDs was relatively low *in vivo*, suggesting that PEG modification can be used to optimize the bioapplication of CuInS_2_ QDs in order to achieve low toxicity.

## Conclusion

In this study, the long-term toxicity of CuInS_2_/ZnS QDs in male mice at a high dose of 25 mg/kg and low dose of 2.5 mg/kg was investigated. Following injection, the CuInS_2_/ZnS QDs accumulated in the liver and spleen over 90 days. There were no obvious changes in body weight or behavior. Routine blood tests and biochemical examination showed that most of the measured parameters were not significantly different between the QDs-treated group and the control group. H&E staining demonstrated that there were no distinct histopathological abnormalities related to treatment with the CuInS_2_/ZnS QDs. In view of these results, we suggest that CuInS_2_/ZnS QDs have high biocompatibility *in vivo* and can be utilized as optical probes or nanocarriers for selected *in vivo* biological applications.

## Ethics Statement

This study was carried out in accordance with the recommendations of ‘the Care and Use of Laboratory Animals Center of Shenzhen University’. The protocol was approved by the ‘Experimental Animal Ethics Committee of Shenzhen University’ (Permit No. 201512022).

## Author Contributions

GL and WZ conceptualized and designed the manuscript. XW and GL supported for administrative. WZ, LL, YC, and DL experimented and collected the data. WZ, ZY, and JW analyzed the data and drafted the manuscript. TC critically revised the manuscript.

## Conflict of Interest Statement

The authors declare that the research was conducted in the absence of any commercial or financial relationships that could be construed as a potential conflict of interest.
