# Supplementary material for: In Vivo Toxicity Evaluation of PEGylated CuInS2/ZnS Quantum Dots in BALB/c Mice
Source: Front Pharmacol. 2019 Apr 25;10:437. doi: 10.3389/fphar.2019.00437 (PMC6497768; doi:10.3389/fphar.2019.00437)
Supplement: Supplementary file 1 [file Table_1.DOCX]

Supplementary Material

**In Vivo Toxicity Evaluation of PEGylated CuInS_2_/ZnS quantum dot in BALB/c mice**

Zou Wenyi^1, 2^ Li Li^2^, Chen Yajing^2^, Chen Tingting^2^, Yang Zhiwen^2^, Wang Jie^2^, Liu Dongmeng^2^, Lin Guimiao^2^*, Wang Xiaomei^1,2^*

^1^ College of Life Science and Oceanography, Shenzhen University, Shenzhen 518060, P. R. China;

^2^ Department of Physiology, School of Basic Medical Sciences, Shenzhen University Health Science Center, Shenzhen University, Shenzhen 518060, P. R. China;

* Correspondence:

Dr Guimiao Lin

Dr Xiaomei Wang ,

xmwang@szu.edu.cn, gmlin@szu.edu.cn

## Supplementary Figures


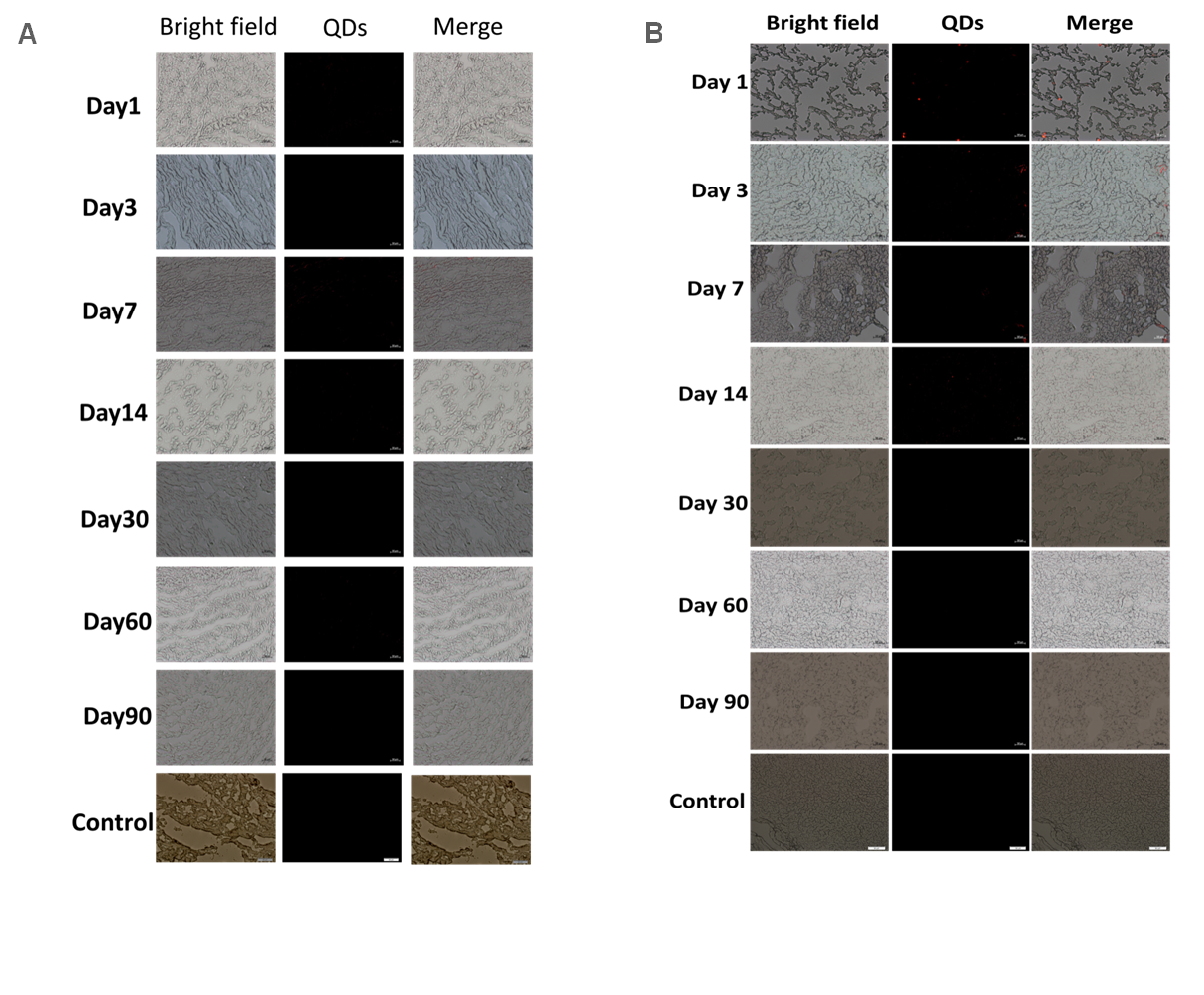


**Supplementary Figure 1.** Fluorescence images of heart (A)and lung (B) of the treated mice at different time points. Scale bar represents 50 μm.


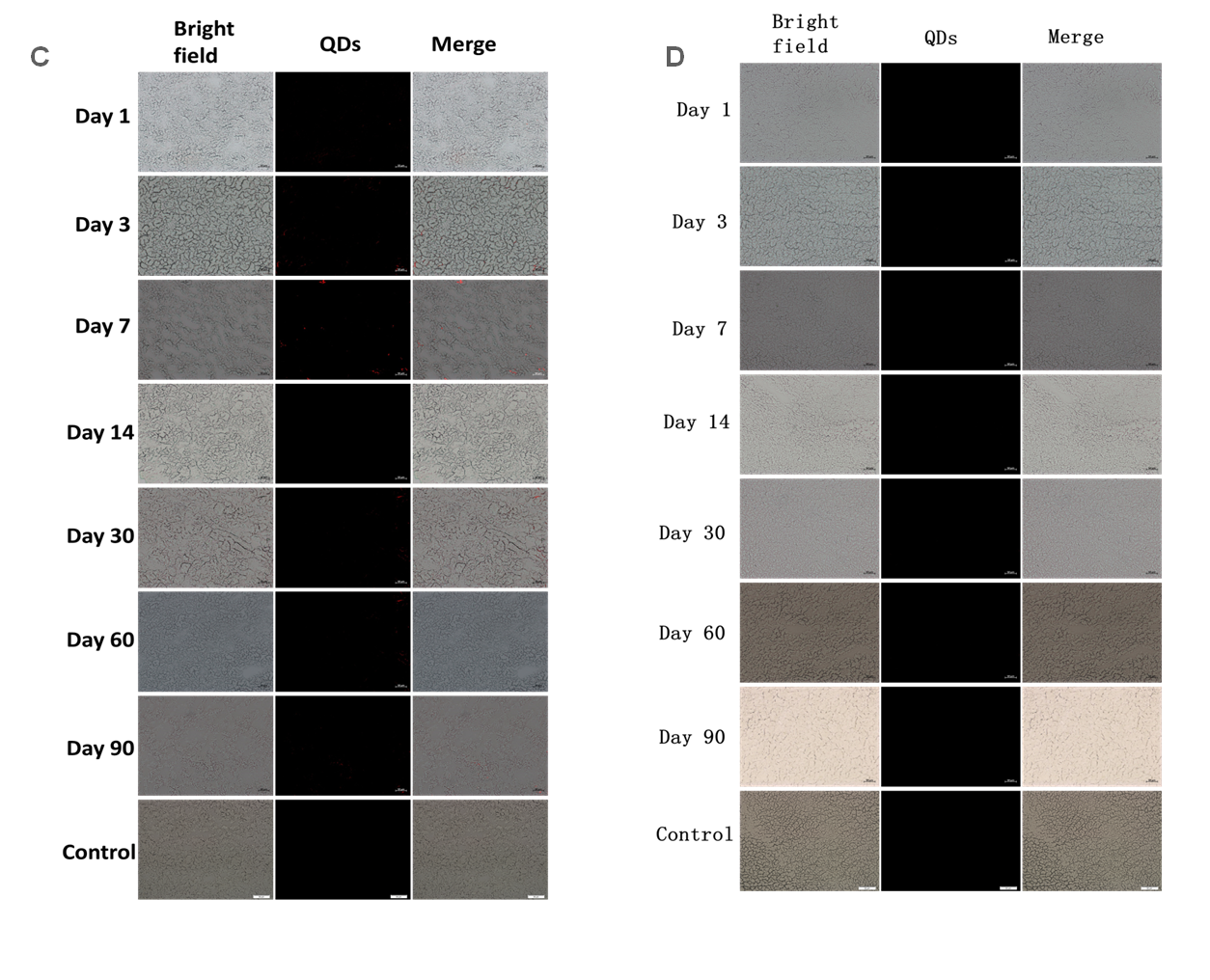


**Supplementary Figure 2.** Fluorescence images of kidney (C) and brain (D) of the treated mice at different time points. Scale bar represents 50 μm.
